# Involving family caregivers in co-design research: a systematic review protocol for developing evidence-based engagement strategies

**DOI:** 10.1136/bmjopen-2025-114457

**Published:** 2026-03-06

**Authors:** Cristina Alfaro-Diaz, Camilla S Rothausen, Emilie V Bonde Hansen, Maria Samuelsson

**Affiliations:** 1 Universidad de Navarra, Pamplona, Navarra, Spain; 2 Department of Oncology, Odense University Hospital, Odense, Denmark; 3 University of Southern Denmark, Odense, Denmark; 4 FaCe—Family focused healthcare research Center, University of Southern Denmark, Odense, Denmark; 5 Department of Care Science, Malmö universitet Fakulteten för hälsa och samhälle, Malmö, Skåne County, Sweden; 6 Child and Family Health Research, Malmö University, Malmö, Sweden

**Keywords:** Caregivers, Research Design, Review

## Abstract

**Abstract:**

**Introduction:**

Stakeholder involvement in research processes is widely recommended to enhance the relevance, quality and uptake of research findings. However, existing studies highlight persistent challenges in engaging family caregivers in co-design research. This gap may result in research outcomes that fail to reflect family caregivers’ needs and preferences, contradicting the core purpose of co-design. Therefore, the aim of this systematic review is to synthesise the available evidence on family caregivers’ experiences of involvement in co-design research and to generate evidence-based strategies to support effective engagement.

**Methods and analysis:**

This systematic review will be conducted using a meta-aggregative approach, following the Joanna Briggs Institute’s (JBI’s) Manual for Evidence Synthesis. Systematic searches will be conducted in PubMed, CINAHL, Scopus, Web of Science and PsycINFO, with no date restrictions. Preliminary searches were performed in EMBASE between September and October 2025. Qualitative primary studies that explore family caregivers’ experiences of involvement in co-design research will be included. Study selection and quality appraisal will be performed independently by two researchers using predefined protocols, disagreements will be resolved through discussion with a third reviewer. After calibration, a single reviewer will extract the data using a customised data extraction template with the dataset distributed among the authors. The first author will then review all extractions. Data will be analysed following JBI’s meta-aggregative method, and results will be presented in narrative summaries, tables and diagrams. The findings will inform strategies for stakeholder involvement in future co-design research. Family caregivers and co-design researchers will be involved in reviewing and revising generated recommendations to enhance their relevance and practical utility.

**Ethics and dissemination:**

This protocol does not involve human participants. The findings of this review will be submitted for publication in a peer-reviewed journal and presented at relevant scientific conferences and meetings.

**PROSPERO registration number:**

CRD420251229190.

**STRENGTHS AND LIMITATIONS OF THIS STUDY**

- The search strategies were developed in collaboration with an experienced research librarian, ensuring methodological rigour and comprehensiveness.
- The inclusion of studies published in English, Spanish, Swedish, Norwegian and Danish broadens the scope and international applicability of the review.
- The complexity and length of the search string may limit replicability and affect the reproducibility of the review process.
- The application of a rigorous, well-established methodological framework will support the production of a high-quality and trustworthy review.
- Family caregivers and co-design researchers will be involved in reviewing and revising generated recommendations to enhance their relevance and practical utility.

## Introduction

 Co-design and participatory approaches, which actively involve stakeholders in the research and development process, are increasingly recommended across disciplines.^1^ These approaches are defined as collaborative processes in which researchers and stakeholders establish a partnership, with stakeholders taking an active role in the research process—from formulating or prioritising research questions to planning data collection, analysing results and disseminating findings.^2 3^ Such methods are recognised for enhancing the relevance, quality and uptake of research,^1^,^3^ as well as for improving recruitment, response and retention rates.^4^ Consequently, conducting research ‘with’ rather than ‘on’ people has become an established principle of contemporary research ethics and practice.^5^,^8^

Despite broad recognition of its value, a previous scoping review on co-design of family interventions in cancer care concluded that involving family caregivers in co-design research remains challenging.^9^ These challenges can result in the marginalisation of caregivers’ perspectives, potentially biasing results toward the views of other stakeholder groups. As a result, research outcomes may not adequately reflect or address family caregivers’ needs and preferences, thereby contradicting the fundamental goals of co-design research. Identifying practical and effective approaches to meaningfully involve family caregivers is, therefore, a pressing priority.

Previous research on family caregivers has shown that, despite context and diagnosis-specific experience, challenges and prerequisites, there are also similarities.^10^,^12^ These include, for instance, that family caregivers tend to prioritise the ill person’s needs over their own; difficulties balancing family caregiving with overall family life, everyday life and work; and the perception of family caregiving as simultaneously meaningful and potentially burdensome.^13^ These are all experiences that may affect the prerequisites for family caregivers’ involvement in co-design research. To advance the research field of co-design, this systematic review aims to reach beyond the context-specific focus of, for instance, technologies and people living with dementia^14^ or mental health or substance use resources/interventions^15^ with the purpose of identifying shared experiences from involvement to guide general recommendations. Correspondingly, for this review, we have applied an inclusive definition of *family caregiver* with ‘family’ and ‘family caregivers’ used interchangeably, defined by their relationship with a patient rather than level of involvement in care. This is further corroborated by conceptual overlaps and inconsistencies between these concepts^16^ and by research highlighting that family caregivers’ level of involvement may change across the illness trajectory^10 12^. Consequently, here, ‘family’ is defined broadly to include any group of two or more individuals connected by strong emotional bonds, a sense of belonging and a commitment to mutual involvement, regardless of blood or social ties.^17^ This includes synonyms such as family members, informal carers, relatives and next of kin. However, due to the specific needs of children,^18^ this review focuses on adult family caregivers of adult patients.

Accordingly, this systematic review aims to synthesise the available evidence on family caregivers’ experiences of involvement in co-design research, with the ultimate goal of developing evidence-based strategies to support their participation. Preliminary searches were conducted in PubMed, PsycINFO, CINAHL, the Cochrane Library and PROSPERO to identify comparable published or ongoing systematic reviews. No such reviews were identified.

## Aim

The objective of this systematic review is to synthesise the available evidence on family caregivers’ experiences of involvement in co-design research and to generate evidence-based strategies to support effective engagement.

## Methods and analysis

The planned review is designed as a qualitative systematic review employing a meta-aggregative approach as outlined by the Joanna Briggs Institute (JBI).^19^ Its design is informed by a prior scoping review that examined key methods and processes for co-designing family interventions in cancer.^9^ That review highlighted the need to establish the evidence base and identify knowledge gaps regarding strategies for supporting family caregiver involvement. The meta-aggregative method was chosen because it enables the synthesis of qualitative evidence to provide a comprehensive and in-depth understanding of a phenomenon, leading to the generation of new, actionable insights.^11^

This protocol follows the Preferred Reporting Items for Systematic Review and Meta-Analysis Protocols (PRISMA-P) checklist,^12^ and the final review will be reported in accordance with the PRISMA guidelines.^13^ The study protocol is registered in PROSPERO (available from https://www.crd.york.ac.uk/PROSPERO/view/CRD420251229190.)

### Research questions

The main research question guiding this review is: *What are the family caregivers’ experiences of being involved in co-design research?*

Specific research questions include:

- What are the barriers and facilitators to family caregivers’ involvement in co-design research?
- What is known about and where are the gaps in understanding how family caregivers perceive their roles, contributions and influence within co-design processes?
- What strategies or conditions promote meaningful and sustained involvement of family caregivers in co-design research?

Following recommendations for meta-aggregation,^20^ a revised Population, Phenomenon of Interest, Context mnemonic will be used to clarify the research question and guide the search strategy (table 1).

**Table 1:** Revised Joanna Briggs Institute Population, Phenomenon of Interest, Context mnemonic for meta-aggregations

Element	Description
Population	Family caregivers
Phenomenon of interest	Experiences of involvement
Context	Co-design research

### Identifying relevant studies

A search strategy was designed in collaboration with an experienced research librarian, informed by the *Peer Review of Electronic Search Strategies Guideline Statement*^21^ and following the *JBI’s Manual for Evidence Synthesis*.^19^ Between September and October 2025, preliminary searches were conducted in EMBASE (see online supplemental appendix 1) to explore relevant keywords and refine the search strategy.

The final search strategy will be applied to the following databases: PubMed, CINAHL, Scopus, Web of Science and PsycINFO. Search terms and concepts will be adapted to each database using appropriate tools, such as Medical Subject Headings, thesaurus terms and Boolean operators, to ensure both sensitivity and specificity. No restrictions will be placed on publication dates.

To ensure comprehensiveness, backward citation searching (reference list screening) and forward citation tracking will be performed for all studies included after full-text screening to identify additional relevant literature. Systematic and scoping reviews identified during the search will be retained for hand-searching their reference lists.

### Eligibility criteria

Studies will be included if they meet the following criteria:

#### Study design

Qualitative, peer-reviewed primary studies published in English, Spanish, Swedish, Norwegian or Danish.

#### Population

Family caregivers, defined as individuals invited to participate in a research project due to their relationship with a patient.^17^ This includes synonyms such as family members, relatives and next of kin.

#### Phenomenon of interest

Experiences of involvement in co-design or participatory research.

#### Context

Co-design and participatory research processes at any stage—planning, conducting or disseminating research. In this review, involvement in co-design refers to a structured collaboration in which researchers and stakeholders form an equitable partnership, and stakeholders actively participate in one or more research activities.^2^

### Study selection

All search results collected from the electronic databases will be exported to Covidence software (Available at www.covidence.org) for screening and data management. The selection of studies will be executed in a three-step manner. First, duplicate studies will be removed. Second, two researchers will independently review the titles and abstracts of all articles to determine eligibility. If discrepancies are present, a third researcher will be involved. In the third step, full-text articles will be reviewed independently by the same two reviewers using predefined inclusion criteria. A third reviewer will be consulted to resolve any remaining disagreements. The final manuscript will include the reasons for excluding articles after full-text review. The study selection process will be presented in a PRISMA flow diagram.

### Data extraction

Following JBI recommendations,^19^ a customised data extraction template will be developed to capture key information, including study references, characteristics, design, methods and participant details. The template will be pilot-tested on three articles by CA, CSR, EVBH and MS to assess its clarity, usefulness and ensure consistency in extraction. After calibration, data from each study will be extracted by a single reviewer, with the dataset distributed among the authors. The first author will then review all the extracted data for completeness and accuracy before analysis.

### Quality appraisal

The JBI Critical Appraisal Checklist for Qualitative Studies will be used to assess the quality of the included studies. First, to ensure alignment among the researchers when interpreting and applying the appraisal tool, the researchers will pilot-test it on two articles before beginning formal appraisal. Each included study will be assessed independently by two reviewers, followed by discussion until consensus is achieved. A third reviewer, experienced in qualitative research, will be available to resolve discrepancies. All studies meeting the inclusion criteria will be retained regardless of quality, as the aim of this review is to capture the full range of available evidence.

### Synthesis

Data synthesis will follow the JBI meta-aggregative approach.^19^ This process involves identifying findings from primary studies, grouping them into categories based on similarity of meaning and synthesising these categories into overarching findings that can inform practice and policy. The first and last authors will conduct the initial analysis, identifying commonalities, differences and preliminary categories. These will be reviewed by a third author after reading all extracted data. The final synthesis and generation of recommendations will be achieved through iterative discussions among all authors.

To enhance credibility,^19^ approximately 3–5 family caregivers engaged in a healthcare service advisory board, and 3–5 co-design researchers, will be invited to participate in consultations to review and revise preliminary recommendations for relevance and practical utility. Potential participants will be informed (verbally and in writing) about the consultation. Family caregivers will be informed by the organisation’s chair during a board meeting, and co-design researchers will be informed via email using snowball sampling within research networks. Those who express interest will be presented with the preliminary findings and recommendations (either face to face or digitally, based on their preferences) and asked to provide verbal feedback.

During the consultations, members of the research team will document their assessments of each recommendation in writing. The results of the consultations may lead to revisions of the recommendations or confirm them as they stand. If major revisions are suggested, the experts will be invited to participate in an additional consultation to assess the revised version(s). The consultation process will be reported in the final study, both in the methods section and as an integrated component of the results.

### Reflexivity

All authors are nurses with expertise in family nursing and qualitative research. CA and MS have specific experience in co-design research involving family caregivers. To minimise potential bias stemming from the researchers’ own perspectives, the team engaged in reflexive discussions to articulate and document their preunderstanding of the phenomenon. These discussions revealed a shared understanding of family caregivers as positioned in the shadow of the patient’s illness, as outlined in the introduction. Hence, our preunderstanding is that family caregivers’ experiences—including perceived barriers and facilitators—are shaped by the practical and emotional consequences of caregiving, which may be both meaningful and burdensome. Reflexivity will be maintained throughout the review process through continuous individual and group reflection. Preliminary analyses will be critically reviewed in light of these reflexive discussions to ensure rigour and transparency.

## Discussion

This systematic review protocol lays the groundwork for a comprehensive exploration of family caregivers’ experiences in co-design research. A key strength of the planned review is its adherence to well-recognised recommendations and transparent reporting, which supports rigour, reproducibility and trustworthiness. The use of a meta-aggregative approach enables the systematic synthesis of qualitative evidence into actionable strategies, while planned consultation with family caregivers and co-design researchers further strengthens credibility by ensuring that the findings are grounded in stakeholder perspectives and practice relevant.

Several limitations should also be acknowledged. Although the search strategy is designed to be comprehensive, the inclusion of multiple synonyms and related terms may result in redundancy and overlap, and the complexity of the search string may pose challenges for exact replication. In addition, despite the inclusion of studies published in a broad range of languages, the review will not encompass all possible languages (eg, French or Mandarin), and relevant evidence published in excluded languages may therefore be missed.

Another limitation concerns the review’s intentionally broad, generic focus on family caregivers’ experiences of involvement in co-design across contexts. Such experiences may be influenced by factors such as illness trajectories, treatment modalities and the availability of formal and informal support systems, which vary across settings. Consequently, the synthesised findings may not be directly transferable to all contexts. Nevertheless, given the existing literature indicating shared and recurring experiences among family caregivers, the review is expected to generate insights that can inform general recommendations, which may be adapted to specific populations and local care contexts.

## Patient and public involvement

Patients and/or the public were not involved in the design, conduct, reporting or dissemination plans of this research protocol. In the forthcoming systematic review, however, family caregivers and co-design researchers will be involved in reviewing and revising generated recommendations to ensure their relevance and practical utility.

## Ethics and dissemination

This protocol does not involve human participants. However, in the forthcoming systematic review, family caregivers engaged in an advisory board and co-design researchers will be consulted as experts. We will not collect any personal, identifiable or sensitive data from participants. Accordingly, under the Swedish Ethical Review Act (2003:460), ethical approval is not required for the consultation. Nevertheless, all participants will receive verbal or written information about the study’s purpose and procedures, and they will be informed that participation is voluntary. All the participants will give verbal or written consent before participating in the consultation. The findings will be submitted for publication in a peer-reviewed journal and presented at relevant scientific conferences and meetings.

## Supplementary material

10.1136/bmjopen-2025-114457 online supplemental file 1
