# Supplementary material for: Involving family caregivers in co-design research: a systematic review protocol for developing evidence-based engagement strategies
Source: BMJ Open. 2026 Mar 6;16(3):e114457. doi: 10.1136/bmjopen-2025-114457 (PMC12970087; doi:10.1136/bmjopen-2025-114457)
Supplement: online supplemental file 1 [file bmjopen-16-3-s001.docx]

**Appendix I.** The search strategy used in EMBASE November 2025

| Table 1. Search profile for EMBASE | |
| --- | --- |
| Set Search Statement | Set Search Statement |
| #1 | (((((((((((((("Caregivers"[Mesh]) OR ("Family"[Mesh])) OR (caregivers[Title/Abstract])) OR (family[Title/Abstract])) OR (families[Title/Abstract])) OR (mothers[Title/Abstract])) OR (mother[Title/Abstract])) OR (fathers[Title/Abstract])) OR (father[Title/Abstract])) OR (carers[Title/Abstract])) OR (carer[Title/Abstract]))) OR (relatives[Title/Abstract])) OR (relative[Title/Abstract])) OR (next of kin[Title/Abstract]) AND |
| #2 | (((((((("Community-Based Participatory Research"[Mesh]) OR (Community-Based Participatory Research[Title/Abstract])) OR (citizen science[Title/Abstract])) OR (participatory research[Title/Abstract])) OR (co-researcher[Title/Abstract])) OR (coresearcher[Title/Abstract])) OR (co-researchers[Title/Abstract])) OR (coresearchers[Title/Abstract])) OR (((((((((((((co-design*[Title/Abstract]) OR (codesign*[Title/Abstract])) OR (participa* design*[Title/Abstract])) OR (co-creat*[Title/Abstract])) OR (cocreat*[Title/Abstract])) OR (co-produc*[Title/Abstract])) OR (coproduc*[Title/Abstract])) OR ((stakehold*[Title/Abstract]) AND (((engag*[Title/Abstract]) OR (involv*[Title/Abstract])) OR (collaborat[Title/Abstract])))) OR ((consumer*[Title/Abstract]) AND (((engag*'[Title/Abstract]) OR (involv*[Title/Abstract])) OR (collaborat*[Title/Abstract])))) OR ((participant[Title/Abstract]) AND (((engag*[Title/Abstract]) OR (involv*[Title/Abstract])) OR (collaborat*[Title/Abstract])))) OR (patient and public involv*[Title/Abstract])) OR (public contribut*[Title/Abstract])) AND (((((((("Research"[Mesh]) OR ("Clinical Trials as Topic"[Mesh])) OR ("Research Personnel"[Mesh])) OR (research[Title/Abstract])) OR (clinical trial*[Title/Abstract])) OR (clinical study[Title/Abstract])) OR (clinical studies[Title/Abstract])) OR (science[Title/Abstract]))) AND |
| #3 | ((((((((((((((("Attitude"[Mesh]) OR (Attitude[Title/Abstract])) OR (experience[Title/Abstract])) OR (experiences[Title/Abstract])) OR (perception[Title/Abstract])) OR (perceptions[Title/Abstract])) OR (perspective[Title/Abstract])) OR (perspectives[Title/Abstract])) OR (view[Title/Abstract])) OR (views[Title/Abstract])) OR (opinion[Title/Abstract])) OR (opinions[Title/Abstract])) OR (reflection[Title/Abstract])) OR (reflections[Title/Abstract])) OR (insight[Title/Abstract])) OR (insights[Title/Abstract]) |
| #4 | 1 AND 2 AND 3 |
| #5 | #4 and Filters: No restrictions were placed on publication dates. Languages included: English, Spanish, Swedish, Norwegian, and Danish |
