# Supplementary material for: Involving family caregivers in co-design research: a systematic review protocol for developing evidence-based engagement strategies
Source: BMJ Open. 2026 Mar 6;16(3):e114457. doi: 10.1136/bmjopen-2025-114457 (PMC12970087; doi:10.1136/bmjopen-2025-114457)
Supplement: Supplementary file 2 [file bmjopen-2025-114457.reviewer_comments.pdf]

PEER REVIEW HISTORY

BMJ Open publishes all reviews undertaken for accepted manuscripts. Reviewers are asked to complete a checklist review form and are provided with free text boxes to elaborate on their assessment. These free text comments are reproduced below.

ARTICLE DETAILS

Title (Provisional)

Involving family caregivers in co-design research: A systematic review protocol for developing evidence-based engagement strategies

Authors

Alfaro, Cristina; Rothausen, Camilla S; Bonde Hansen, Emilie V; Samuelsson, Maria

---

VERSION 1 - REVIEW

Reviewer 1

Name Hoddinott, Graeme

Affiliation The University of Sydney, School of Public Health, Faculty of Medicine and Health

Date 13-Jan-2026

COI None

Congratulations to the co-authors on a well-written manuscript addressing an important topic. Some suggestions for refinement:

Line 133 (and paragraph overall) - please define 'family caregivers' here in the background section. Are they parents of children? Healthcare providers delivering formal care to elderly? Etc. And if this is very inclusive (i.e., any type of family caregiver) then you would need to explain how literature on different types of caregivers can be synthesized, e.g., will you present findings by type?

Lines 151-155 - please elaborate on why a scoping review was not selected instead. Your logic is that the literature on the topic is limited, and scoping reviews are recommended in that instance (minor tweak).

Lines 167-168 - suggest instead: "What is known about and where are the gaps in understanding how family caregivers perceive their roles ..." since this will provide you with greater flexibility in how you present findings (especially if literature is sparce).

Line 177 - past tense rather?

Line 242 - how will these experts be solicited?

Lines 250-252 - could you articulate now already what some of your expected findings might be? To show us what your preunderstandings of the phenomenon are. This protocol paper review can be part of the reflexivity.

Lines 256-284 - consider shortening overall. There is a lot of repetition. And this is a protocol paper, so there cannot be 'conclusions' from it or many points of discussion.

Final paragraphs - consider instead include a more explicit description of the strengths and limitations. Noting that while the range of languages you include is impressively broad, it doesn't include every language (e.g., French, Mandarin) - this is just a limitation to be acknowledged, not a critique. Also, see earlier point on 'family caregivers', I would imagine that what is / counts as 'family caregiving' can be very context / resource dependent, and some articulation that the definition used may not apply universally would be useful.

VERSION 1 - AUTHOR RESPONSE

Congratulations to the co-authors on a well-written manuscript addressing an important topic. Some suggestions for refinement:

Line 133 (and paragraph overall) - please define 'family caregivers' here in the background section. Are they parents of children? Healthcare providers delivering formal care to elderly? Etc. And if this is very inclusive (i.e., any type of family caregiver) then you would need to explain how literature on different types of caregivers can be synthesized, e.g., will you present findings by type?

Author response: Thank you for highlighting the need for clarification. We have addressed this in the introduction. Please see changes in red font on lines 135-155.

Lines 151-155 - please elaborate on why a scoping review was not selected instead. Your logic is that the literature on the topic is limited, and scoping reviews are recommended in that instance (minor tweak).

Author response: Thank you for this insightful comment. We have addressed the need for a more distinct rationale for the chosen design. Please see changes in red font on lines 173-177.

Lines 167-168 - suggest instead: "What is known about and where are the gaps in understanding how family caregivers perceive their roles ..." since this will provide you with greater flexibility in how you present findings (especially if literature is sparce).

Author response: Thank you very much for this suggestion, which enhanced the manuscript!

Please see the changes in red on lines 192-193.

Line 177 - past tense rather?
Author response: Yes, of course. Thank you for bringing this to our attention. Please see changes in red font on lines 198.

Line 242 - how will these experts be solicited?
Author response: We thank the reviewer for this comment. Family caregivers will be solicited via the Swedish patient and next-of-kin board: the organization's chair will inform board members both verbally and in writing during a board meeting. Co-design researchers will be invited via email using snowball sampling within relevant research networks. Those who express interest will be invited to digital meetings to review the preliminary findings and strategies and provide feedback on their accuracy, relevance, and applicability. Please see the changes in red on lines 273-281.

Lines 250-252 - could you articulate now already what some of your expected findings might be? To show us what your preunderstandings of the phenomenon are. This protocol paper review can be part of the reflexivity.

Author response: This is a great idea! Please find the expanded reflexivity section in red font on lines 287-292.

Lines 256-284 - consider shortening overall. There is a lot of repetition. And this is a protocol paper, so there cannot be 'conclusions' from it or many points of discussion.
Author response: This has been addressed. Please see the changes in the revised section in red font on lines 298-320.

Final paragraphs - consider instead include a more explicit description of the strengths and limitations. Noting that while the range of languages you include is impressively broad, it doesn't include every language (e.g., French, Mandarin) - this is just a limitation to be acknowledged, not a critique.

Author response: This has been addressed. Please see the revised section in red font on lines 306-311.

Also, see earlier point on 'family caregivers', I would imagine that what is / counts as 'family caregiving' can be very context / resource dependent, and some articulation that the definition used may not apply universally would be useful.

Author response: Again, thank you for this insightful comment! This has been addressed. Please see changes in red font on lines 313-320.
